# Predicting the Unpredictable: AI-Driven Prognosis in Pancreatic Neuroendocrine Neoplasms

**DOI:** 10.3390/cancers18020306

**Published:** 2026-01-19

**Authors:** Elettra Merola, Emanuela Pirino, Stefano Marcucci, Franca Chierichetti, Andrea Michielan, Laura Bernardoni, Armando Gabbrielli, Maria Pina Dore, Giuseppe Fanciulli, Alberto Brolese

**Affiliations:** 1 Dipartimento di Medicina, Chirurgia e Farmacia, University of Sassari, Viale San Pietro 43, 07100 Sassari, Italympdore@uniss.it (M.P.D.); gfanciu@uniss.it (G.F.); 2 Hepato-Pancreato-Biliary (HPB) Unit, Department of General Surgery, Santa Chiara Hospital, Azienda Provinciale per i Servizi Sanitari (APSS), 38122 Trento, Italy; stefano.marcucci@apss.tn.it (S.M.); alberto.brolese@apss.tn.it (A.B.); 3 Department of Nuclear Medicine, Santa Chiara Hospital, Azienda Provinciale per i Servizi Sanitari (APSS), 38122 Trento, Italy; 4 Gastroenterology and Digestive Endoscopy Unit, Santa Chiara Hospital, Azienda Provinciale per i Servizi Sanitari (APSS), 38122 Trento, Italy; andrea.michielan@apss.tn.it (A.M.); laura.bernardoni@apss.tn.it (L.B.); armando.gabbrielli@apss.tn.it (A.G.); 5 Center for Medical Sciences (CISMed), University of Trento, 38122 Trento, Italy; 6 Baylor College of Medicine, One Baylor Plaza Blvd, Houston, TX 77030, USA

**Keywords:** pancreatic neuroendocrine neoplasms, artificial intelligence, prognostic models, survival, clinical outcomes

## Abstract

The clinical management of Pancreatic Neuroendocrine Neoplasms (Pan-NENs) is complicated by the disease’s intrinsic variability, which creates significant hurdles for accurate risk profiling and the standardization of treatment protocols. Recently, Artificial Intelligence (AI) has offered a promising avenue to address these challenges. By integrating and processing high-dimensional multimodal datasets (encompassing clinical history, radiomics, and pathology), these computational tools can refine survival forecasts and support the development of personalized medicine. However, the transition from experimental success to routine clinical use is currently obstructed by reliance on limited, retrospective cohorts that lack external validation, alongside unresolved concerns regarding algorithmic transparency and ethical governance. This review evaluates the current landscape of AI-driven prognostic modeling for Pan-NENs and critically examines the pathway towards their reliable integration into clinical practice.

## Simple Summary

Pancreatic Neuroendocrine Neoplasms (Pan-NENs) represent a unique challenge in oncology due to their varied nature, making accurate risk stratification and treatment selection challenging. Artificial Intelligence (AI) has emerged as a powerful tool capable of mining extensive clinical and diagnostic data to refine predictions regarding patient survival and metastatic spread. Despite enthusiastic early results, the field faces hurdles, including ethical dilemmas and a lack of large-scale, validated studies. This review investigates the current landscape of AI-based predictive tools and their effectiveness in forecasting clinical outcomes for Pan-NEN patients.

## 1. Introduction

Pancreatic neuroendocrine neoplasms (Pan-NENs) constitute a rare category of pancreatic malignancies, representing roughly 1–5% of all pancreatic tumors. Despite their rarity, epidemiological data derived from population-based registries indicate a steady increase in their reported incidence over the last decades. This phenomenon is largely attributed to advances in diagnostic pathways, including widespread use of high-resolution cross-sectional imaging, increased access to functional nuclear medicine modalities, and heightened clinical awareness of neuroendocrine diseases, rather than to a genuine rise in disease occurrence [1].

Pan-NENs are characterized by marked biological and clinical heterogeneity. Their clinical presentation ranges from small, slowly progressive lesions identified incidentally to highly aggressive neoplasms with rapid growth and early dissemination, with metastatic disease documented in up to 50% of patients at initial diagnosis [1]. As a result, curative surgical resection is achievable in only a limited proportion of cases [2]. For most patients, management follows a prolonged and complex course, requiring the careful sequencing of systemic therapies over time. In tumors expressing somatostatin receptors, treatment options include long-acting somatostatin analogues (SSAs) and peptide receptor radionuclide therapy (PRRT) [3,4], while additional strategies encompass targeted agents and cytotoxic chemotherapy. In recognition of this complexity, international societies such as the European Neuroendocrine Tumor Society advocate for care within specialized multidisciplinary centers, where individualized therapeutic planning, structured surveillance, and timely treatment adaptation can be ensured [5,6,7].

Key determinants of prognosis in Pan-NENs include the World Health Organization (WHO) classification [8]—that distinguishes between pancreatic well-differentiated neuroendocrine tumors (Pan-NETs) and poorly differentiated neuroendocrine carcinomas (Pan-NECs)—the Ki-67 proliferation index, and the extent of disease at presentation [9]. The prognostic models most frequently adopted in clinical research are based on Cox proportional hazards regression. While these tools offer valuable insights, they explain only a proportion of the heterogeneity observed in clinical outcomes. Traditional statistical models may overlook the complex, often non-linear interactions among clinical variables, tumor biology, imaging phenotypes, and treatment patterns. In this context, artificial intelligence (AI)-based methodologies have gained increasing attention. Machine learning (ML) and deep learning (DL) techniques enable the analysis of high-dimensional data and may uncover patterns that are not readily identifiable using traditional analytical frameworks. However, the current evidence base for AI applications in Pan-NENs remains methodologically constrained. Many studies involve small sample sizes, heterogeneous gastro-entero-pancreatic neuroendocrine populations, and retrospective single-center datasets, with limited use of rigorous external validation. Additional unresolved issues include model interpretability, reproducibility, and the establishment of robust ethical and governance standards [10,11].

This narrative review provides a critical and focused synthesis of AI-driven prognostic modeling in Pan-NENs. Its distinct contribution is anchored in three deliberately chosen and interlocking dimensions, designed to address specific gaps in the extant literature. First, it imposes a strict temporal boundary, synthesizing evidence published exclusively within the most recent five-year period (2021–2025), thereby capturing the most contemporary and technically sophisticated methodological advances. Second, it deliberately restricts its clinical scope to sporadic Pan-NENs, the most prevalent yet prognostically heterogeneous subtype, ensuring a coherent and clinically actionable analysis that is not confounded by the distinct pathophysiology of genetic syndromes. Part of the observations summarized in this review had been previously reported [10,11].

## 2. Methods

An updated literature search was performed to identify original studies investigating the use of AI techniques in Pan-NENs. The search strategy combined terms related to AI methodologies with neuroendocrine tumor nomenclature, using the following query: (“artificial intelligence” OR AI OR “deep learning” OR “machine learning”) AND (neuroendocrine AND (tumor OR carcinoma OR neoplasm)) [10].

The search was limited to articles published between 1 January 2021, and November 1, 2025, and retrieved 755 papers. Eligibility criteria are summarized in Table 1. Studies were included if they reported primary data on AI-driven models developed to predict prognostic outcomes in patients with Pan-NENs. Publications not involving gastro-entero-pancreatic NENs (*n* = 454) were excluded, as were review articles, editorials, and commentaries (*n* = 67), as well as studies in which AI did not constitute a core analytical component (*n* = 107). An additional 113 papers were excluded because they did not report prognostic outcomes specific to Pan-NENs or did not focus on prognosis. Ultimately, 12 studies met the inclusion criteria and were included in the present review. Only the PubMed database was searched, in line with the narrative nature of the review.

To ensure completeness, the reference lists of all eligible studies were manually screened to identify additional relevant publications that met the inclusion criteria but were not captured by the initial database search.

AI-based tools were used to assist with language editing, stylistic refinement, and figure preparation (ChatGPT-4o, OpenAI; Google Gemini 3). Responsibility for study design, data interpretation, and scientific conclusions remains entirely with the authors.

## 3. AI-Driven Prognostic Models for Pan-NENs

Recent developments in AI-based prognostic assessment have largely concentrated on survival prediction [12,13,14,15,16,17,18,19,20,21,22,23]. A summary of the publications included in this review is presented in Table 2 and Table S1. Reported results refer to the (external) validation cohort, when available.

### 3.1. Prediction of Overall Survival

Li et al. [12] analyzed a large population-based cohort comprising 1998 patients with Pan-NETs identified within the Surveillance, Epidemiology, and End Results (SEER) registry. The dataset was partitioned into development and validation subsets and further supplemented by an independent external cohort derived from a Chinese population. Model construction followed a stepwise hybrid framework integrating classical statistical techniques with machine-learning methodologies. Feature selection was performed using Least Absolute Shrinkage and Selection Operator (LASSO) regression, followed by the application of multiple predictive approaches, including random forest algorithms, logistic regression, and Cox proportional hazards models. The final prognostic model achieved a concordance index of 0.76 for overall survival (OS), indicative of moderate discriminatory performance. Nonetheless, the interpretability and generalizability of these findings are limited by several factors, including the retrospective nature of the analysis, incomplete capture of clinically relevant variables within the SEER database, the limited testing beyond the development dataset, and the absence of integrated radiomic or molecular data.

Another study by Jiang et al. [13] explored the prognostic potential of a DeepSurv neural network to estimate 5 and 10-year OS in a cohort of 3239 patients with Pan-NENs extracted from the SEER registry. The cohort was randomly divided into training and testing sets using a 7:3 ratio. The DeepSurv architecture was trained on routinely available clinical parameters, such as age at diagnosis, tumor grade, surgical treatment, chemotherapy exposure, and indicators of locoregional disease extension. When benchmarked against conventional Cox regression, neural multitask logistic regression, and random survival forest models, DeepSurv consistently demonstrated superior discrimination, achieving area under the curve (AUC) values ranging from 0.87 to 0.90 for long-term survival prediction. To facilitate clinical translation, the model was implemented as an open-access web-based calculator. On the other hand, the absence of confirmation in external populations, together with limited treatment granularity and lack of imaging or genomic inputs, constrains confidence in its applicability outside the development cohort.

Focusing specifically on advanced disease, Yu et al. [14] developed a ML survival model specifically tailored to patients with metastatic Pan-NETs, leveraging SEER data from 1430 individuals. Following dimensionality reduction using a random survival forest-driven feature selection strategy, ten clinically relevant variables were retained for model development. Seven distinct survival algorithms were subsequently constructed and compared. Among these, eXtreme Gradient Boosting (XGBoost) exhibited the most robust performance, with time-dependent AUC values of 0.78, 0.75, and 0.74 at 1, 3, and 5 years, respectively. Tumor grade, surgical management, nodal status, and metastatic burden emerged as dominant predictors. The authors further operationalized the model through an interactive online interface to support individualized prognostication. Despite these strengths, reliance on retrospective registry data, absence of Ki-67 labeling indices and treatment response metrics, and lack of external multicenter validation remain significant methodological limitations.

In contrast to population-based registry analyses, Singh et al. [15] conducted a large single-center retrospective study focusing on 447 patients with metastatic Pan-NENs treated with PRRT. The authors developed the PANEN-N model using Random Survival Forests to identify 17 key prognostic variables encompassing clinical, biochemical, imaging, and pathological parameters. The model demonstrated strong discriminatory capability, with concordance indices ranging from 0.82 to 0.86 in internally validated analyses. A dynamic web-based nomogram was created to facilitate individualized survival counseling in clinical practice. While this study represents one of the most comprehensive PRRT-specific prognostic tools currently available, its retrospective single-center design and absence of external validation limit its broader generalizability.

Finally, Hillman et al. [16] investigated whether unsupervised ML-based clustering could enhance prognostic stratification beyond the conventional American Joint Committee on Cancer (AJCC) TNM staging system. Using data from 3225 Pan-NET cases in the SEER registry, the authors applied the Ensemble Algorithm for Clustering Cancer Data (EACCD), an approach that groups patients based on survival dissimilarities rather than predefined stage boundaries. When restricted to TNM variables, the algorithm generated four prognostic clusters with improved separation of Kaplan–Meier survival curves compared with AJCC staging, yielding a modestly higher concordance index (0.6685 vs. 0.6656). Incorporation of age at diagnosis resulted in five distinct prognostic groups and further improved discrimination (C-index 0.7015), producing clearer survival gradients. Although this study underscores the limitations of committee-based staging systems and highlights the potential of data-driven clustering for refined risk stratification, important challenges remain, including small subgroup sizes, lack of biological or radiomic features in SEER, and the absence of clearly defined internal or external validation strategies required for clinical implementation.

### 3.2. Prediction of Recurrence-Free Survival and Metastases

In the area of recurrence forecasting, Murakami et al. [17] examined recurrence-free survival (RFS) in a multicenter cohort of 371 patients with G1–G2 Pan-NETs who had undergone curative-intent resection across 22 Japanese institutions between 1987 and 2020. By applying a Random Survival Forest approach, the authors demonstrated improved prognostic discrimination compared with conventional Cox regression, with AUC values ranging from 0.73 to 0.83. Notably, the model captured non-linear effects of Ki-67 and identified tumor diameter exceeding 20 mm as a dominant driver of recurrence risk. Despite these results, the extended enrollment window spanning more than three decades represents a relevant source of potential bias, given substantial temporal changes in imaging modalities, histopathological classification, and therapeutic strategies. Moreover, although internal data splitting into training and testing sets was performed, the lack of external validation limits the robustness of these findings.

A different multimodal strategy was explored by Ma et al. [18], who focused on postoperative hepatic recurrence in a retrospective series of 163 patients treated with radical surgery. The investigators integrated heterogeneous inputs into a composite nomogram, achieving exceptionally high discriminative performance, with reported AUC values approaching unity. Stratification based on the model output separated patients into distinct recurrence-risk categories, with substantially shorter median RFS observed in the high-risk group. Despite these encouraging results, concerns regarding generalizability remain substantial, as the analysis was constrained by a limited sample size, variability in surgical approaches, and the absence of validation in independent external cohorts.

In the field of imaging-based prognostic assessment, Song et al. [19] developed a pipeline aimed at estimating the probability of 5-year recurrence following surgical resection. Their methodology combined automated lesion segmentation using a U-Net architecture with high-level feature extraction through a convolutional neural network applied to arterial-phase CT images. The radiomics signature achieved moderate-to-good discrimination in external validation, with AUC values between 0.77 and 0.83, and showed additional performance gains when supplemented with a small number of clinical variables. Although derived from a retrospective dataset of limited size, this work illustrates how the integration of quantitative imaging features with minimal clinical information can enhance individualized, non-invasive perioperative risk assessment.

An ultrasound-based approach was proposed by Huang et al. [20], who constructed a prognostic framework integrating contrast-enhanced ultrasound (CEUS) features with conventional clinical variables in 72 patients with Pan-NENs. Using a convolutional neural network pretrained on a large natural-image dataset and adapted to CEUS imaging, the authors demonstrated the feasibility of semi-automatic lesion segmentation and reported moderate discriminatory ability for recurrence prediction after curative surgery. This approach capitalizes on the ability of CEUS to characterize tumor microvascular perfusion, a surrogate marker increasingly linked to biological aggressiveness. In contrast, the study’s retrospective design, small cohort size, and limited validation beyond the development dataset underscore the need for further confirmatory investigations.

Regarding metastatic risk, Bi and Yu [21] analyzed a large population-based cohort of 7463 Pan-NET cases from the SEER registry to develop an interpretable ML model for predicting synchronous liver metastases. Following feature selection procedures, a limited set of clinicopathological variables was retained, and multiple algorithms were compared. Gradient boosting methods demonstrated the highest discriminative accuracy in the validation cohort, and the resulting model was translated into an online calculator to facilitate clinical use. Despite its strong performance, the reliance on registry data without therapy details, biological or imaging information, together with the absence of external validation, constrains its immediate applicability.

Increasing attention is being directed toward prognostic models that incorporate genomic and transcriptomic data to refine risk assessment in Pan-NENs. By interrogating molecular alterations that drive tumor behavior, these approaches aim to delineate biologically meaningful subgroups and improve the precision of disease monitoring. Unlike imaging-derived models, which primarily reflect macroscopic or phenotypic characteristics, molecular analyses offer insight into the underlying pathways that fuel heterogeneity across tumors.

In this setting, Greenberg et al. [22] explored whether gene-expression profiles from resected Pan-NETs could discriminate tumors with high versus low metastatic propensity. Using a ML framework, the authors identified a concise transcriptomic signature (AURKA, CDCA8, CPB2, MYT1L, NDC80, PAPPA2, SFMBT1, ZPLD1) that achieved high discriminative accuracy and retained performance in an independent validation cohort analyzed with a clinically applicable platform. These findings support the premise that molecular profiling at the time of surgery may refine postoperative risk stratification and inform surveillance intensity or early systemic treatment considerations. However, the high costs of sequencing, specialized infrastructure requirements, and incomplete integration with established clinical workflows remain significant obstacles to widespread adoption.

A complementary proteogenomic perspective was provided by Ji et al. [23], who performed an integrated multi-omic analysis of 108 treatment-naïve Pan-NETs and validated their findings in an external cohort. By combining genomic, transcriptomic, and proteomic data within a unified prognostic framework, the authors identified molecular signatures capable of stratifying patients according to recurrence risk (AUC: 0.96–0.98), with clear separation of survival outcomes between high and low-risk groups. While these results highlight the potential of multi-omic integration to refine postoperative risk assessment, their translational impact is tempered by limited cohort sizes, incomplete clinical annotation, and the relatively small number of cases available for validation. As such, although high-risk patients identified by these models may be candidates for intensified surveillance strategies, broader validation in larger, clinically annotated populations is required before routine implementation.

## 4. Discussion

**Figure 1:**
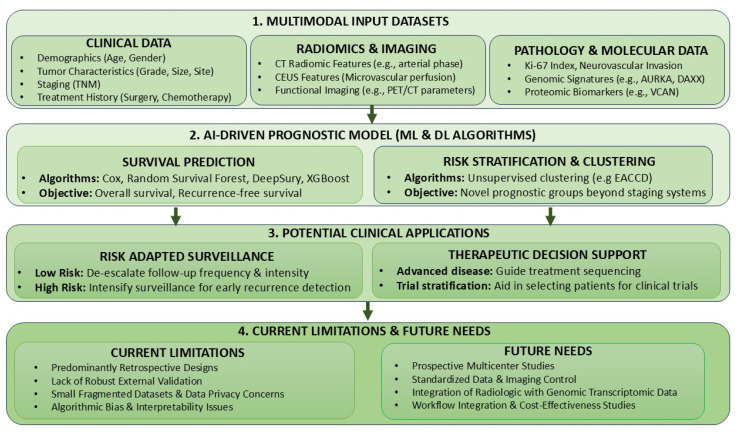
AI-driven prognostic modeling in Pan-NENs: from multimodal data to clinical application. CT: Computed Tomography; CEUS: Contrast-Enhanced Ultrasound; PET: Positron Emission Tomography; AURKA: Aurora Kinase A; DAXX: Death Domain Associated Protein; VCAN: Versican Core Protein; ML: Machine Learning; DL: Deep Learning; XGBoost: eXtreme Gradient Boosting; EACCD: Ensemble Algorithm for Clustering Cancer Data.

AI is progressively influencing how Pan-NENs are evaluated and managed, with increasing evidence that it can enhance diagnostic accuracy, refine prognostic stratification, and support individualized therapeutic decisions. The literature summarized in this review demonstrates that AI research in Pan-NENs spans a broad methodological spectrum (Figure 1). However, despite encouraging performance metrics, the evidence base supporting routine clinical implementation remains immature.

A substantial proportion of published models relies on large population-based registries [12,13,14,21]. These datasets offer the advantage of broad patient representation and statistical power, enabling the development of survival and metastasis-prediction models with apparent generalizability. In contrast, institution-based studies [15,16,17,18,20,21,22,23] typically integrate clinical, pathological, imaging, or molecular data, allowing more detailed modeling of tumor behavior. However, across both settings, the predominance of retrospective designs, limited transparency in data preprocessing, and the frequent absence of external validation remain major obstacles to translation into clinical practice. High discrimination alone does not equate to clinical utility; prognostic tools must ultimately demonstrate that they can meaningfully inform decision-making and improve patient-centered outcomes.

From a clinical perspective, several hypothetical but clinically plausible applications of validated AI-based prognostic models can be envisioned. In the post-resection setting, accurate estimation of absolute recurrence risk could support risk-adapted surveillance strategies. Patients predicted to have a very low probability of recurrence at predefined time points might reasonably undergo de-escalation of follow-up intensity, reducing radiation exposure, healthcare costs, and patient burden. Conversely, individuals classified as high risk could benefit from intensified surveillance protocols and earlier detection of potentially treatable relapse.

In advanced or metastatic disease, integrated prognostic tools may also assist in therapeutic sequencing. For example, nomograms combining clinical, laboratory, and imaging variables to estimate expected benefit from PRRT could support multidisciplinary discussions when selecting among PRRT, targeted therapies, or chemotherapy in borderline-risk scenarios. Such tools may also facilitate identification of patients most suitable for enrollment in clinical trials evaluating emerging treatments.

Importantly, these scenarios remain conceptual. They are presented not as clinical recommendations but as a framework illustrating how AI-derived prognostic stratification could complement existing management paradigms. Transitioning from statistically promising models to clinically trusted decision-support systems will require prospective validation, demonstration of impact on patient outcomes, cost-effectiveness analyses, and seamless integration into clinical workflows—benchmarks that have not yet been met.

### 4.1. Methodological Pitfalls in ML Prognostic Modeling

Several technical challenges recurrently affect ML-based prognostic studies in Pan-NENs and warrant explicit discussion. One major concern is data leakage, which can occur when information from the test set inadvertently influences model training, feature selection, or hyperparameter tuning. This risk is particularly relevant in single-center imaging and radiomics studies [18,19,20,22,23], where feature extraction and selection are often performed before data splitting, potentially inflating reported performance.

Closely related is the issue of overfitting and optimism, especially in small datasets with high-dimensional feature spaces. Many institutional studies report strong discrimination despite limited sample sizes and a large number of candidate predictors, increasing the likelihood that models capture noise rather than true signal. Although internal validation strategies such as cross-validation are frequently employed, these approaches do not fully substitute for independent external validation, which remains scarce across the reviewed literature.

Class imbalance represents another critical limitation, particularly for metastatic or recurrence endpoints. In registry-based studies predicting synchronous liver metastases or long-term survival [12,13,14,21], the proportion of events may be relatively low compared with non-events, biasing models toward majority classes and potentially reducing sensitivity for clinically relevant high-risk patients. Only a minority of studies explicitly report strategies to address imbalance, such as resampling techniques or cost-sensitive learning.

Handling of missing data is inconsistently reported. Large registries frequently lack key variables, including Ki-67, treatment sequencing, or response data, while institutional cohorts may have incomplete imaging or molecular annotations. Exclusion of cases with missing values, a common but often underreported practice, may introduce selection bias and further limit generalizability.

Finally, label heterogeneity poses a particularly relevant challenge in Pan-NEN research. Several studies span long inclusion periods, during which WHO classifications, grading thresholds, and pathological criteria have evolved. As a result, outcome labels and predictor definitions may not be consistent over time, introducing systematic noise into model training and potentially impairing reproducibility. This issue is amplified in pooled or registry-based analyses, where reclassification according to contemporary standards is often not feasible.

Taken together, these methodological vulnerabilities highlight the need for greater rigor in study design, transparent reporting of data preprocessing and validation strategies, and closer alignment with emerging methodological standards for AI research in oncology. Addressing these challenges will be essential to ensure that future AI-based prognostic models in Pan-NENs are not only statistically robust but also clinically credible and reproducible.

### 4.2. Ethical and Structural Considerations

Ethical considerations further complicate clinical translation. AI development depends on access to large and diverse datasets [24,25], raising questions regarding data ownership, governance, sharing practices, and the adequacy of patient consent for secondary data use. Only a minority of oncology-focused AI studies—including those involving NEN cohorts—provide access to training datasets or clearly describe consent procedures [26,27], limiting transparency and potentially undermining trust, particularly when commercial reuse of data is involved.

Algorithmic bias remains an additional concern [28,29]. Under-representation of specific demographic or clinical groups during model development may lead to systematic errors when models are applied more broadly. In rare diseases such as Pan-NENs, small cohort sizes and uneven data contributions across centers further exacerbate this risk. Bias-mitigation strategies, including diverse multicenter datasets, subgroup performance analyses, and fairness reporting, are therefore essential.

In Pan-NENs specifically, data scarcity increases privacy risks and contributes to “health-data poverty,” whereby rare-disease populations benefit less from data-driven innovation [26]. These constraints underscore the importance of privacy-preserving approaches such as federated learning and of coordinated, ethically grounded collaborative research frameworks [30]. Without such efforts, progress in this field is likely to remain fragmented and incremental, limiting the clinical impact of otherwise promising AI methodologies.

## 5. Conclusions

In conclusion, this review provides an updated overview of the current literature addressing the application of AI-based models for prognostic prediction in Pan-NENs. Although several studies encouraging predictive performance, their clinical relevance remains uncertain. If appropriately validated, AI-driven tools may contribute to more personalized management by optimizing surveillance strategies—such as reducing follow-up intensity in patients at low risk of recurrence or progression—and by supporting treatment selection according to disease aggressiveness.

From a methodological perspective, meaningful progress will depend on coordinated, multicenter prospective studies. These efforts should incorporate harmonized imaging protocols, standardized clinical endpoints, integration of radiologic data with genomic and transcriptomic profiling, and systematic documentation of therapeutic exposure and sequencing. In parallel, health–economic evaluations and workflow integration studies are required to assess real-world feasibility. Importantly, successful implementation will also require sustained ethical oversight, regulatory clarity, and institutional commitment. With these prerequisites in place, next-generation prognostic models could deliver dynamic, patient-specific risk estimates and support a more precise and individualized management paradigm for patients with Pan-NENs.

## Figures and Tables

**Table 1:** Predefined eligibility criteria for literature search.

**Inclusion criteria**
Original research articles
Focused on AI applications for sporadic Pan-NEN prognosis
Published between 2021 and 2025
**Exclusion criteria**
Studies not directly involving both AI and Pan-NENs
Review articles (narrative or systematic)
Editorials, research commentaries, or opinion pieces
Conference abstracts

AI: Artificial Intelligence; Pan-NEN: Pancreatic Neuroendocrine Neoplasm.

**Table 2:** AI-based prognostic models for predicting survival and metastases in Pan-NENs.

Study	Study Population	Objective	AI Model	Variables Included in the Analysis	Results	Validation	Calibration Assessment
OS							
Hillman 2025 [16]	SEER-based, 3225 Pan-NENs	Prediction of OS	EACCD	AJCC TNM system, age	- C-index: 0.70	Not reported	Not reported
Jiang 2023 [13]	SEER-based, 3239 Pan-NENs	Prediction of 5- and 10-yr OS	DeepSurv neural network vs. NMTLR, Random Survival Forest, Cox model	Age, gender, race, marital status, primary site, grade, tumor size, tumor extension, treatment	- Best performance by DeepSurv - AUC: 0.87 (5-yr) 0.90 (10-yr) - Web calculator provided	- Internal: train-test split + five-fold cross validation on training dataset - External: missing	Calibration curve + Integrated Brier Score
Li 2023 [12]	SEER-based, 1998 Pan-NETs + 245 Chinese cases	Prediction of OS	LASSO + Random-Forest feature selection → logistic and Cox nomogram models	Diagnostic model: grade, N-stage, surgery, chemotherapy, tumor size, bone metastasis Prognostic model: subtype, grade, surgery, age, brain metastases	- Nomogram outperforms TNM staging system - C-index: 0.76	- Internal: train-test split - External: retrospective series	Calibration curve (bootstrapping)
Singh 2025 [15]	Single-center, 447 Pan-NETs after PRRT	Prediction of OS	Random Survival Forest, Cox model	Age, gender, grade, Karnofsky performance score, weight loss, tumor functionality, time from diagnosis to first PRRT, hepatomegaly, Hedinger syndrome, metastatic pattern, lab values, [^18^F]FDG-PET/CT positivity	- C-index: 0.82–0.86	- Internal: train-test split - External: missing	Not reported
Yu 2025 [14]	SEER-based, 1430 metastatic Pan-NETs	Prediction of OS	Seven ML-based prognostic models	Age, gender, primary site, TNM, tumor grade, surgery, chemotherapy,	- Best performance: XGBoost algorithm - AUC: 0.74 (5-yr)	- Internal: train-test split - External: missing	Calibration curve
			RFS and metastatic risk				
Bi 2025 [21]	SEER-based, 7463 Pan-NETs	Prediction of liver metastases	Combination of LASSO and Boruta, 10 ML algorithms	Age, gender, race, marital status, social status, TNM, size, functional status, primary site, grade, metastatic pattern, surgery	- Best performance GBM - AUC: 0.91	-Internal: train-test split + ten-fold cross validation on training dataset -External: missing	Calibration curve
Greenberg 2024 [22]	Multicenter, 95 Pan-NETs	Prediction of metastatic recurrence	ML applied to define a transcriptomic-based gene panel	Genes: SV2, chromogranin A and B, (*TPH1*), *ARX*, *PDX1*, *UCHL1*, novel 8-gene panel (*AURKA*, *CDCA8*, *CPB2*, *MYT1L*, *NDC80*, *PAPPA2*, *SFMBT1*, *ZPLD1*)	- AUC: 0.88	- Internal: train-test split + “leave-one-out” cross validation on training dataset - External: matched testing cohort of 29 patients	Not reported
Huang 2021 [20]	Single-center, 72 Pan-NENs	Prediction of 3-yr RFS	Semiautomatic segmentation DL method applied to CEUS: Fine-tuned SE-ResNeXt-50 CNN + multivariate logistic nomogram	CEUS images, arterial enhancement level, tumor size	- AUC: 0.78	- Internal: train-test split + five-fold cross validation on training dataset - External: missing	Calibration curve + Hosmer–Lemeshow goodness-of-fit test
Ji 2025 [23]	Single-center, 108 Pan-NETs + 51 external validation cohort	Prediction of RFS	Reproducible Prognosis Molecular Signature platform (ML-based model)	Proteogenomic data, data about disease recurrence	- Identified three-protein prognostic signature (GNAO1, INA, VCAN)	- Internal: train-test split - External: missing	Calibration curve
Ma 2024 [18]	Single-center, 163 Pan-NETs	Prediction of RFS	Integrated nomogram (Pathomics logistic score + ResNet-based DLR + nerve infiltration)	Gender, age, tumor site in the pancreas, vascular/nerve infiltration, stage, ATRX/DAXX, Ki-67 hotspot index, MH index, DLR score	- AUC: 0.96 (median RFS) - C-index: 0.96	- Internal: train-test split + ten-fold cross validation on training dataset - External: missing	Calibration curve (bootstrapping) + Hosmer–Lemeshow goodness-of-fit test
Murakami 2023 [17]	Multicenter, 371 Pan-NETs G1/G2	Prediction of RFS	Random Survival Forest vs. Cox model	Ki-67, WHO grade, tumor size, residual tumor status, lymph node metastases	- Best performance by Random Survival Forest - AUC: 0.73–0.83 (5-yr) - C-index: 0.84	- Internal: train-test split - External: missing	Calibration curve + Hosmer–Lemeshow goodness-of-fit test
Song 2021 [19]	Multicenter, 56 Pan-NENs	Prediction of 5-yr RFS	U-Net segmentation + DL radiomics (SE-ResNeXt-50) + SVM	Age, neuroendocrine symptoms, arterial-phase DLR features	- AUC: 0.77–0.83	- Internal: ten-fold cross validation - External: 18 patients	Not reported

AI: Artificial Intelligence; OS: Overall Survival; SEER: Surveillance, Epidemiology, and End Results; Pan-NEN: Pancreatic Neuroendocrine Neoplasm; EACCD: Ensemble Algorithm for Clustering Cancer Data; AJCC: American Joint Committee on Cancer; C-index: Concordance Index; NMTLR: Neural Multi-Task Logistic Regression; AUC: Area Under the Curve; Pan-NET: Pancreatic Neuroendocrine Tumor; LASSO: Least Absolute Shrinkage and Selection Operator; PRRT: Peptide Receptor Radionuclide Therapy; [^18^F]FDG-PET/CT: 18 F-Fluorodeoxyglucose Positron Emission Tomography; ML: Machine Learning; XGBoost: eXtreme Gradient Boosting; GBM: Gradient Boosting Machine; RFS: Recurrence-free Survival; DL: Deep Learning; CEUS: Contrast-Enhanced Ultrasound; CNN: Convolutional Neural Network; GNAO1: Guanine Nucleotide-binding Protein Subunit Alpha; INA: Alpha-Internexin; VCAN: Versican Core Protein; DLR: Deep Learning-Radiomics; MH: Morisita–Horn; WHO: World Health Organization; SVM: Support Vector Machine.

## Data Availability

No new data were created or analyzed in this study. Data sharing is not applicable to this article.

## Supplementary Materials

The following supporting information can be downloaded at: https://www.mdpi.com/article/10.3390/cancers18020306/s1, Table S1: Interpretability and code availability of AI-based prognostic models in Pan-NENs.

## Abbreviations

The following abbreviations are used in this manuscript:

Pan-	Pancreatic
NEN	Neuroendocrine Neoplasm
NET	Neuroendocrine Tumor
SSTR	Somatostatin Receptor
SSA	Somatostatin Analog
PRRT	Peptide Receptor Radionuclide Therapy
WHO	World Health Organization
AI	Artificial Intelligence
ML	Machine Learning
DL	Deep Learning
SEER	Surveillance, Epidemiology, and End Results
LASSO	Least Absolute Shrinkage and Selection Operator
C-index	Concordance Index
OS	Overall Survival
AUC	Area Under the Curve
RFS	Recurrence-Free Survival
CT	Computed Tomography
AJCC	American Joint Committee on Cancer
EACCD	Ensemble Algorithm for Clustering Cancer Data
CEUS	Contrast-Enhanced Ultrasound
GBM	Gradient Boosting Machine
CNN	Convolutional Neural Network
NMTLR	Neural Multi-Task Logistic Regression
DLR	Deep Learning-Radiomics
[^18^F]FDG-PET/CT	18F-fluorodeoxyglucose positron emission tomography
SVM	Support Vector Machine
XGBoost	eXtreme Gradient Boosting
