# Supplementary material for: Predicting the Unpredictable: AI-Driven Prognosis in Pancreatic Neuroendocrine Neoplasms
Source: Cancers (Basel). 2026 Jan 19;18(2):306. doi: 10.3390/cancers18020306 (PMC12839224; doi:10.3390/cancers18020306)
Supplement: Supplementary file 1 [file cancers-18-00306-s001.zip › cancers-4076433-supplementary.pdf]

Table S1. Interpretability and code availability of AI-based prognostic models in Pan-NENs

Study	Interpretability approach	Code/online tool availability
	**OS**	
Hillman 2025 [1]	Survival-based clustering	Yes (public code; modified for this study)
Jiang 2023 [2]	Permutation-based feature importance	Yes (web-based calculator)
Li 2023 [3]	Feature selection via LASSO; variable importance from ML models	NR
Singh 2025 [4]	Variable importance from Random Survival Forest	Yes (web-based nomogram)
Yu 2025 [5]	SHAP-based feature importance	Yes (web-based calculator)
	**RFS and metastatic risk**	
Bi 2025 [6]	Feature selection (Boruta, LASSO); model-based variable importance	Yes (web-based calculator)
Greenberg 2024 [7]	Gene-expression signature (feature selection–based)	NR
Huang 2021 [8]	Nomogram coefficients (clinical + DL probability)	NR
Ji 2025 [9]	Proteogenomic feature selection; nomogram-based risk stratification	NR
Ma 2024 [10]	Multivariable nomogram (clinical + imaging features)	NR
Murakami 2023 [11]	Random Survival Forest variable importance; partial dependence plots	No (code explicitly not shared)
Song 2021 [12]	Radiomics/DL feature selection; risk stratification	NR

AI: Artificial Intelligence; Pan-NENs: Pancreatic Neuroendocrine Neoplasms; OS: Overall Survival; LASSO: ML: Machine Learning; NR: Not Reported; SHAP: SHapley Additive exPlanations; RFS: Recurrence-free Survival; DL: Deep Learning

1. Hillman, J.; Clark, Q.; Rehm, L.; Ahmed, A.E.; Chen, D. Using Machine Learning to Revise the AJCC Staging System for Neuroendocrine Tumors of the Pancreas. *Cancers* **2025**, *17*, 3658. https://doi.org/10.3390/cancers17223658.
2. Jiang, C.; Wang, K.; Yan, L.; Yao, H.; Shi, H.; Lin, R. Predicting the survival of patients with pancreatic neuroendocrine neoplasms using deep learning: A study based on Surveillance, Epidemiology, and End Results database. *Cancer Med.* **2023**, *12*, 12413–12424. https://doi.org/10.1002/cam4.5949.
3. Li, J.; Huang, L.; Liao, C.; Liu, G.; Tian, Y.; Chen, S. Two machine learning-based nomogram to predict risk and prognostic factors for liver metastasis from pancreatic neuroendocrine tumors: A multicenter study. *BMC Cancer* **2023**, *23*, 529. https://doi.org/10.1186/s12885-023-10893-4.
4. Singh, A.; Sanduleanu, S.; Kulkarni, H.R.; Langbein, T.; Lambin, P.; Baum, R.P. The PANEN nomogram: Clinical decision support for patients with metastatic pancreatic neuroendocrine neoplasm referred for peptide receptor radionuclide therapy. *Front. Endocrinol.* **2025**, *16*, 1514792. https://doi.org/10.3389/fendo.2025.1514792.
5. Yu, Z.; Zheng, Y.; Wang, K.; Fang, Z.; Huang, H.; Gao, Z.; Du, C.; Zhang, C.; Huang, D.; Zhang, J.; et al. A clinically applicable machine learning model for personalized survival prediction in metastatic pancreatic neuroendocrine tumors. *Eur. J. Surg. Oncol.* **2025**, *51*, 110222. https://doi.org/10.1016/j.ejso.2025.110222.
6. Bi, J.; Yu, Y. Predicting liver metastasis in pancreatic neuroendocrine tumors with an interpretable machine learning algorithm: A SEER-based study. *Front. Med.* **2025**, *12*, 1533132. https://doi.org/10.3389/fmed.2025.1533132.

7. Greenberg, J.A.; Shah, Y.; Ivanov, N.A.; Marshall, T.; Kulm, S.; Williams, J.; Tran, C.; Scognamiglio, T.; Heymann, J.J.; Lee-Saxton, Y.J.; et al. Developing a Predictive Model for Metastatic Potential in Pancreatic Neuroendocrine Tumor. *J. Clin. Endocrinol. Metab.* **2024**, *110*, 263–274. https://doi.org/10.1210/clinem/dgae380.
8. Huang, B.; Lin, X.; Shen, J.; Chen, X.; Chen, J.; Li, Z.P.; Wang, M.; Yuan, C.; Diao, X.F.; Luo, Y.; et al. Accurate and Feasible Deep Learning Based Semi-Automatic Segmentation in CT for Radiomics Analysis in Pancreatic Neuroendocrine Neoplasms. *IEEE J. Biomed. Health Inform.* **2021**, *25*, 3498–3506. https://doi.org/10.1109/JBHI.2021.3070708.
9. Ji, S.; Cao, L.; Gao, J.; Du, Y.; Ye, Z.; Lou, X.; Liu, F.; Zhang, Y.; Xu, J.; Shi, X.; et al. Proteogenomic characterization of non-functional pancreatic neuroendocrine tumors unravels clinically relevant subgroups. *Cancer Cell* **2025**, *43*, 776–796.E14. https://doi.org/10.1016/j.ccell.2025.03.016.
10. Ma, M.; Gu, W.; Liang, Y.; Han, X.; Zhang, M.; Xu, M.; Gao, H.; Tang, W.; Huang, D. A novel model for predicting postoperative liver metastasis in R0 resected pancreatic neuroendocrine tumors: Integrating computational pathology and deep learning-radiomics. *J. Transl. Med.* **2024**, *22*, 768. https://doi.org/10.1186/s12967-024-05449-4.
11. Murakami, M.; Fujimori, N.; Nakata, K.; Nakamura, M.; Hashimoto, S.; Kurahara, H.; Nishihara, K.; Abe, T.; Hashigo, S.; Kugiyama, N.; et al. Machine learning-based model for prediction and feature analysis of recurrence in pancreatic neuroendocrine tumors G1/G2. *J. Gastroenterol.* **2023**, *58*, 586–597. https://doi.org/10.1007/s00535-023-01987-8.
12. Song, C.; Wang, M.; Luo, Y.; Chen, J.; Peng, Z.; Wang, Y.; Zhang, H.; Li, Z.P.; Shen, J.; Huang, B.; et al. Predicting the recurrence risk of pancreatic neuroendocrine neoplasms after radical resection using deep learning radiomics with preoperative computed tomography images. *Ann. Transl. Med.* **2021**, *9*, 833. https://doi.org/10.21037/atm-21-25.
